# Demonstration and validation of Kernel Density Estimation for spatial meta-analyses in cognitive neuroscience using simulated data

**DOI:** 10.1016/j.dib.2017.06.003

**Published:** 2017-06-07

**Authors:** Michel Belyk, Steven Brown, Sonja A. Kotz

**Affiliations:** a Faculty of Psychology and Neuroscience, Department of Neuropsychology and Psychopharmacology, University of Maastricht, Maastricht, The Netherlands; b Department of Psychology, Neuroscience & Behaviour, McMaster University, Hamilton, Ontario, Canada; c Department of Neuropsychology, Max Planck Institute for Human and Cognitive Sciences, Leipzig, Germany

**Keywords:** Meta-analysis, Kernel Density Estimation, Activation likelihood estimation, Cognitive neuroscience, Inferior frontal gyrus

## Abstract

The data presented in this article are related to the research article entitled “Convergence of semantics and emotional expression within the IFG pars orbitalis” (Belyk et al., 2017) [1]. The research article reports a spatial meta-analysis of brain imaging experiments on the perception of semantic compared to emotional communicative signals in humans. This Data in Brief article demonstrates and validates the use of Kernel Density Estimation (KDE) as a novel statistical approach to neuroimaging data. First, we performed a side-by-side comparison of KDE with a previously published meta-analysis that applied activation likelihood estimation, which is the predominant approach to meta-analyses in cognitive neuroscience. Second, we analyzed data simulated with known spatial properties to test the sensitivity of KDE to varying degrees of spatial separation. KDE successfully detected true spatial differences in simulated data and displayed few false positives when no true differences were present. R code to simulate and analyze these data is made publicly available to facilitate the further evaluation of KDE for neuroimaging data and its dissemination to cognitive neuroscientists.

## Specifications Table

**Table:** 

Subject area	*Cognitive Neuroscience*
More specific subject area	*Validation of Statistical Method*
Type of data	*Analysis, Figure, Code*
How data was acquired	*Meta-analysis, Simulation*
Data format	*Analyzed, Simulation*
Experimental factors	1) *Affective vs. linguistic prosody* 2) *Simulated spatial locations in the inferior frontal gyrus*
Experimental features	1) *Replication: We used KDE to replicate a previous meta-analysis that used the standard activation likelihood estimation approach.* 2) *Simulation: We used KDE to analyze simulated brain-imaging meta-data with known spatial properties.*
Data accessibility	*The data can be simulated using the R scripts in the supplementary materials of this article.*

## **Value of the data**

• The data provide a means of evaluating Kernel Density Estimation (KDE) as a novel statistical approach to neuroimaging data.
• The R code included with this article will facilitate cognitive neuroscientists in simulating data to perform their own evaluations of KDE and applying it to other datasets.
• KDE allows researchers to restrict analyses to regions of interest in stereotaxic space for the purpose of testing a priori hypotheses without mandatory whole-brain exploratory analyses.
• Implementation in the publicly available R statistical computing language facilitates interfacing KDE with flexible and cutting-edge statistical tools for further methodological development.
• KDE may be computed at a higher spatial resolution than other methods, although at the cost of computational efficiency.

## 1 Data

### 1.1 Demonstration by replication

**Fig. 1:**
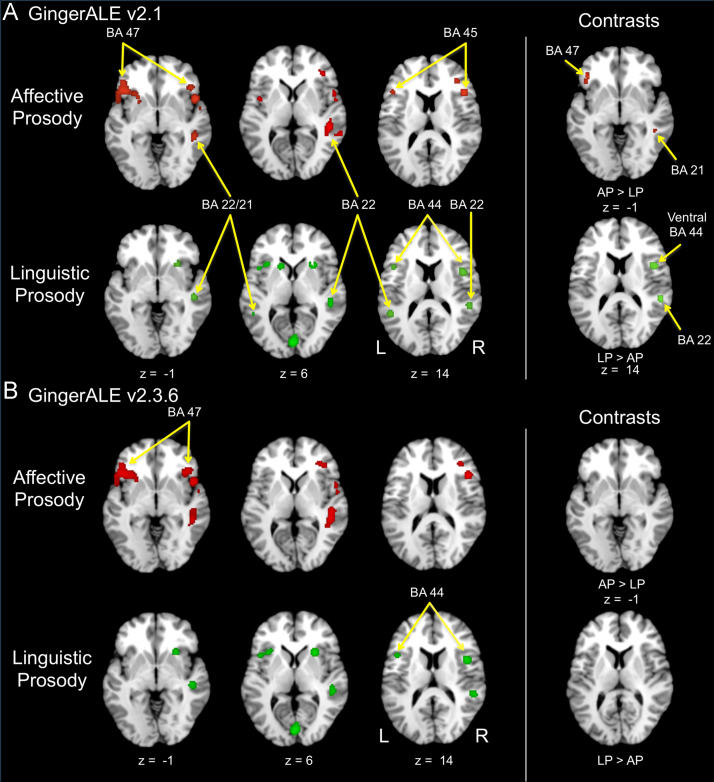
Comparison of GingerALE software versions. A) ALE analysis of affective and linguistic prosody using GingerALE v2.1, as reported in Belyk and Brown [2]. B) Re-analysis of the same data using GingerALE v2.3.6. in light of implementation errors in earlier versions of the software. Left panels show individual results per function of interest, and right panels show contrasts between functions. AP: affective prosody; BA: Brodmann Area; LP: linguistic prosody.

Fig. 1 presents a comparison of Activation Likelihood Estimation (ALE) across GingerALE software versions. GingerALE v2.3.6 detected all major areas of interest from the original analysis, but failed to detect any differences in direct contrasts.

**Fig. 2:**
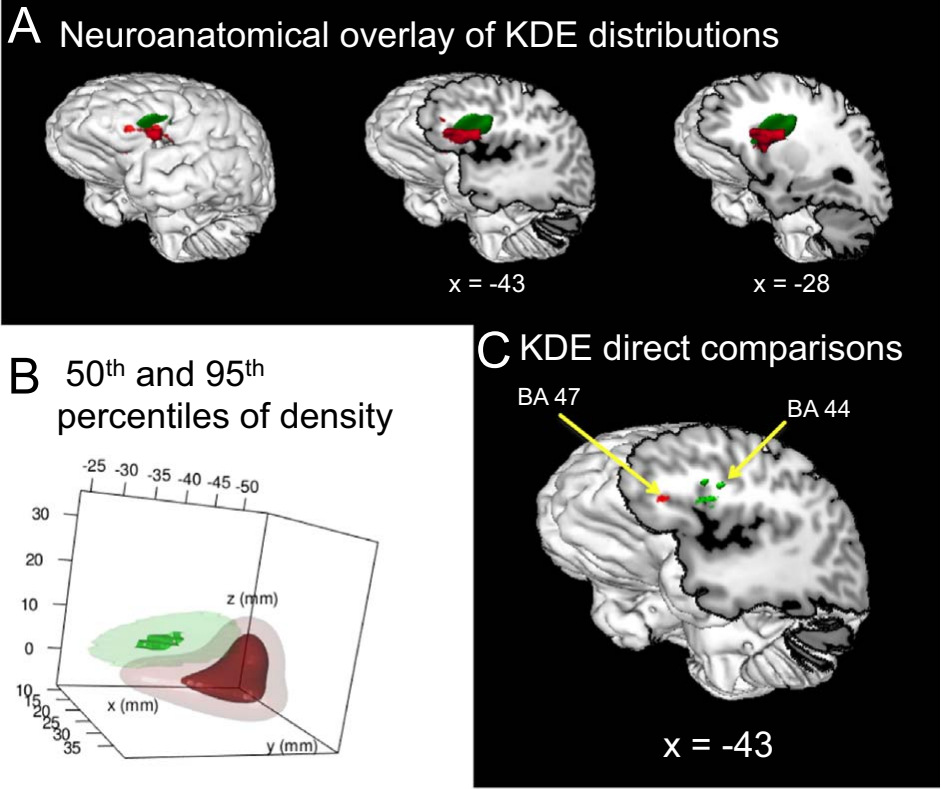
Replication of Belyk and Brown [2] using KDE. A) Volumes of the 95th percentile of density overlaid on a neuroanatomical template in Talairach space for affective prosody (red) and linguistic prosody (green). B) Surfaces representing the 50th (translucent) and 95th (opaque) percentiles of density for affective and linguistic prosody. C) Parametric map of contrasts between affective and linguistic prosody. KDE demonstrated some ability to detect differences between conditions that were not detected by ALE. Further tests would be useful in order to quantify the relative sensitivity of these two approaches and assess the influence of the subsampling procedure for small datasets.

Fig. 2 presents a replication of the same analysis using the KDE approach described in Belyk et al. [1], but restricted to an area of interest in the inferior frontal gyrus (IFG). Localization of linguistic prosody to the IFG pars opercularis was observable using the KDE approach. Affective prosody was localized to the IFG pars orbitalis, but only if sub-sampling procedures were omitted. This may be due to the very small sample size for linguistic prosody.

### 1.2 Demonstration by simulation

**Fig. 3:**
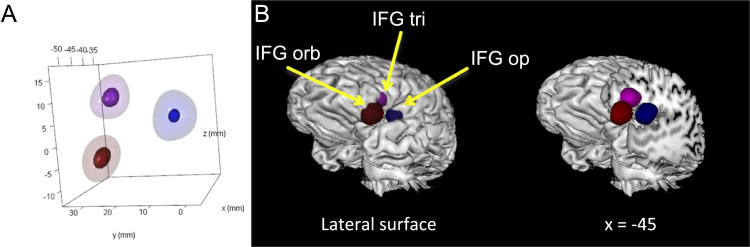
Simulated non-overlapping density distributions around idealized centroids in the IFG pars opercularis (blue), IFG pars triangularis (purple), and IFG pars orbitalis (red). A) The 50th (translucent) and 95th (opaque) percentiles of density for each simulated data set. Axes represent the three cardinal dimensions of space in stereotaxic brain maps. B) Volume of the 95th percentile of density rendered on a neuroanatomical template. IFG: inferior frontal gyrus; Op: pars opercularis; Orb: pars orbitalis: Tri: pars triangularis.

**Fig. 4:**
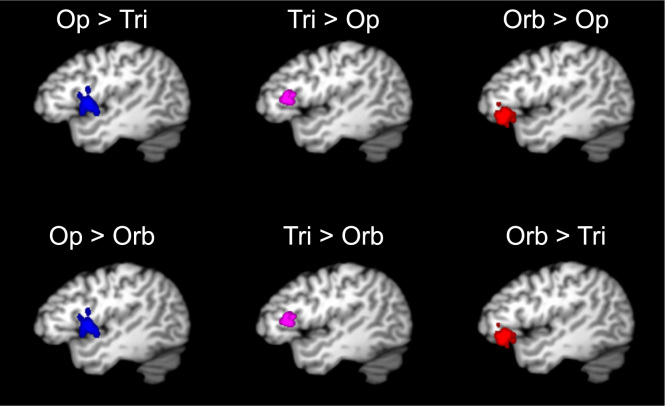
Significance volumes from pairwise comparisons between idealized locations within the IFG pars opercularis (blue), IFG pars triangularis (purple), and IFG pars orbitalis (red) compared to each other region. All slices are at *x* = −45. Op: pars opercularis; Tri: pars triangularis; Orb: pars orbitalis.

Fig. 3 presents density distributions for data simulated around idealized non-overlapping centroids within the three major divisions of the IFG. Fig. 4 presents the results of statistical contrasts between each simulated IFG location. KDE correctly localized each simulated brain area and distinguished each location from the others (cluster sizes ranging from 2392 mm^3^ to 4184 mm^3^).

**Fig. 5:**
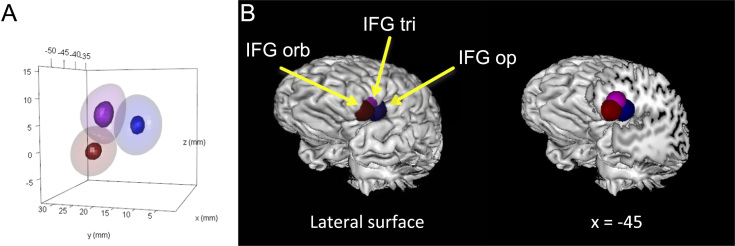
Simulated overlapping density distributions around centroids in the IFG pars opercularis (blue), IFG pars triangularis (purple), and IFG pars orbitalis (red). A) The 50th (translucent) and 95th (opaque) percentiles of density for each simulated data set. Axes represent the three cardinal dimensions of space in stereotaxic brain maps. B) Volume of the 95th percentile of density rendered on a neuroanatomical template. IFG: inferior frontal gyrus; Op: pars opercularis; Orb: pars triangularis: Tri: pars triangularis.

**Fig. 6:**
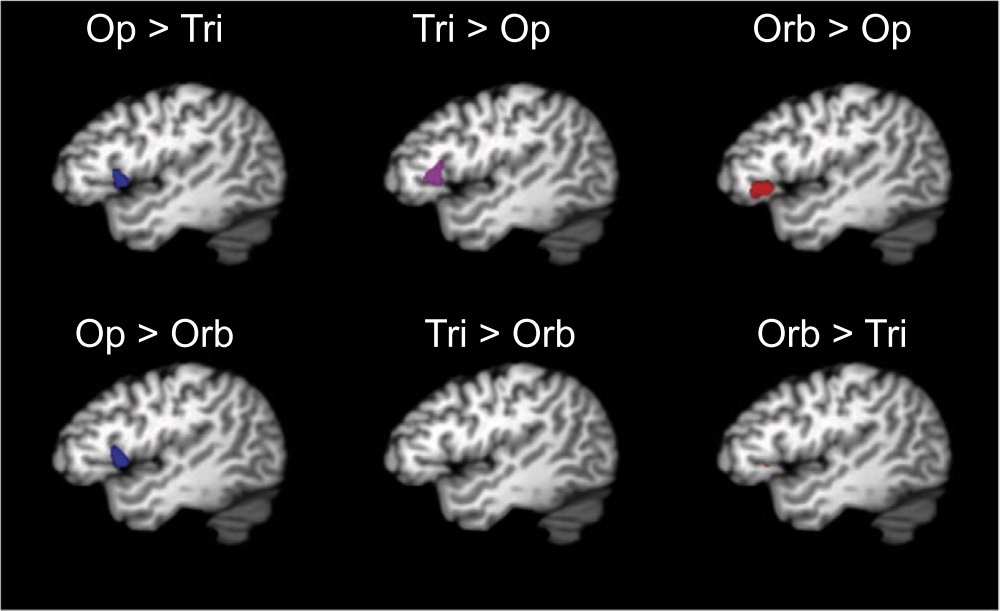
Significance volumes from pairwise comparisons between proximate locations within the IFG pars opercularis (blue), IFG pars triangularis (purple), and IFG pars orbitalis (red) compared to each other region. As expected from the properties of the simulated data, these volumes are more proximate than those observed in Fig. 4. The significant volumes are also smaller, reflecting the greater degree of overlap between simulated density distributions. All slices are at *x* = −45. Op: pars opercularis; Tri: pars triangularis; Orb: pars orbitalis.

Fig. 5 presents partly overlapping density distributions for data simulated around more-proximate centroids. Fig. 6 presents the results of statistical contrasts between these more-proximate simulated locations. The IFG pars opercularis was easily distinguished from the other locations (cluster sizes ranging from 759 mm^3^ to 946 mm^3^). A small volume was significantly denser for the simulated IFG pars orbitalis than for the IFG pars triangularis (cluster size 4 mm^3^). No volume was significantly denser in the reverse contrast.

A null simulation examined the behavior of KDE for data drawn from identical distributions. In three of the six pairwise comparisons, this simulation resulted in at least one false positive voxel. Most clusters were composed of single voxels, although the three largest were 13 mm^3^, 15 mm^3^, and 94 mm^3^ in size, respectively. Clusters of contiguous false-positive voxels are expected considering the spatial smoothness of KDE, and these false-positive clusters do not approach the volumes detected in simulations of non-overlapping locations, simulations of overlapping locations, or the effects reported in Belyk et al. [1].

## 2 Experimental design, materials and methods

### 2.1 Demonstration by replication

We compare the use of KDE with the ALE approach that may be more familiar to cognitive neuroscientists. We first replicated a previous meta-analysis [2], [4] using an updated version of the ALE algorithm (v.2.3.6) [4]. Second, we extracted all of the coordinates of brain activations within the IFG from the original dataset. This yielded 27 coordinates from 11 experiments of affective prosody and 5 coordinates for 5 experiments of linguistic prosody. These data were analyzed using the statistical approach described in Belyk et al. [1].

### 2.2 Demonstration by simulation

We simulated coordinates of brain activations in three-dimensional stereotaxic space from 150 “experiments”. Coordinates were divided equally between the three major divisions of the IFG. These were simulated by sampling points from multivariate normal distributions centered around the following coordinates: −45, 5, 5 (simulating the IFG pars opercularis), −45, 25, 10 (simulating the IFG pars triangularis), and −45, 30, −5 (simulating the IFG pars orbitalis). The shapes of the multivariate normal distributions were modeled [5] after the density distribution observed from the replication experiment reported in Section 1.1, which spanned approximately the same brain space. This distribution had the variance-covariance structure that is provided in the Supplementary data file “sigma.RData”. KDE was used to detect these simulated locations. See Supplementary materials for R code underlying data simulation and analysis.

In order to test the sensitivity of the KDE approach to more-proximate and partially overlapping density distributions, we performed the same analysis on data simulated from distributions that were centered halfway between the idealized locations noted above and a common center of mass between them. These coordinates were: −45, 12.5, 4.2 (simulating the IFG pars opercularis), −45, 22.5, 6.7 (simulating the IFG pars triangularis), and −45, 22.5, 1.25 (simulating the IFG pars orbitalis). Finally, in order to test the selectivity of KDE, we repeated the same analysis on data simulated from a single distribution centered around the coordinate: −45, 20, 3.3.
